# Comparison of the Medical Uses and Cellular Effects of High and Low Linear Energy Transfer Radiation

**DOI:** 10.3390/toxics10100628

**Published:** 2022-10-21

**Authors:** Eric Russ, Catherine M. Davis, John E. Slaven, Dmitry T. Bradfield, Reed G. Selwyn, Regina M. Day

**Affiliations:** 1 Graduate Program of Cellular and Molecular Biology, Uniformed Services University of the Health Sciences, Bethesda, MD 20814, USA; 2 Department of Pharmacology and Molecular Therapeutics, Uniformed Services University of the Health Sciences, Bethesda, MD 20814, USA; 3 Department of Radiology, University of New Mexico, Albuquerque, NM 87131, USA

**Keywords:** ionizing radiation, linear energy transfer, cellular effects, signal transduction, biological effects, transcriptomic analysis

## Abstract

Exposure to ionizing radiation can occur during medical treatments, from naturally occurring sources in the environment, or as the result of a nuclear accident or thermonuclear war. The severity of cellular damage from ionizing radiation exposure is dependent upon a number of factors including the absorbed radiation dose of the exposure (energy absorbed per unit mass of the exposure), dose rate, area and volume of tissue exposed, type of radiation (e.g., X-rays, high-energy gamma rays, protons, or neutrons) and linear energy transfer. While the dose, the dose rate, and dose distribution in tissue are aspects of a radiation exposure that can be varied experimentally or in medical treatments, the LET and eV are inherent characteristics of the type of radiation. High-LET radiation deposits a higher concentration of energy in a shorter distance when traversing tissue compared with low-LET radiation. The different biological effects of high and low LET with similar energies have been documented in vivo in animal models and in cultured cells. High-LET results in intense macromolecular damage and more cell death. Findings indicate that while both low- and high-LET radiation activate non-homologous end-joining DNA repair activity, efficient repair of high-LET radiation requires the homologous recombination repair pathway. Low- and high-LET radiation activate p53 transcription factor activity in most cells, but high LET activates NF-kB transcription factor at lower radiation doses than low-LET radiation. Here we review the development, uses, and current understanding of the cellular effects of low- and high-LET radiation exposure.

## 1. Introduction

The initial discovery of radiation energy occurred in the 1890s, with almost simultaneous reports from Wilhelm Konrad von Rȍntgen, A. Henri Becquerel, and Marie Sklodowska Curie and her husband Pierre Curie (1895–1898) [1,2,3]. Almost immediately following its discovery, radiation began to be put into use for medical imaging as well as for clinical treatments [1,2,3,4]. Ionizing radiation is defined as short wavelength, high-frequency energy produced by natural or artificial sources, that when interacting with matter is capable of producing ions at the molecular level [5]. Exposure to ionizing radiation can occur during medical imaging and treatments such as radiologic imaging, nuclear medicine imaging, and radiotherapy for cancer; from environmental exposures from naturally occurring terrestrial radiation such as radon, cosmic sources, and low-level industrial sources; or from nuclear accidents or nuclear weapons [5,6,7,8,9,10,11]. The use of low- and high-dose, and low- and high-linear energy transfer (LET) radiation is increasing for medical applications, and there is concern regarding high-LET radiation exposure during space travel [1,7,12]. This review explores the different medical uses and biological effects of low- and high- LET radiation.

### Characteristics of Ionizing Radiation

Ionizing radiation comes in a variety of forms including quantized electromagnetic waves, or photons, and atomic and subatomic particles [1,5,12]. Ionizing radiation strips electrons from atoms, resulting in a free electron and a positively charged nucleus, which are known as an ion pair [5]. The effects of radiation exposure and the resulting biological damage are extremely variable, depending upon the amount of energy absorption that occurs in the biological tissue (the absorbed dose, often expressed as joules per kilogram, or gray [Gy]), the dose rate of exposure (Gy per unit time), and the total area or volume of exposure [1,5]. The dose, the dose rate, and the area of exposure can be altered by the amount of radioactive material present, the distance from the radiation source, and the presence of shielding material [1,5,12]. Additionally, radioactive species have unique inherent characteristics, including intrinsic energies, measured in electron volts (eV), and amounts of energy that they transfer when passing through matter, measured as linear energy transfer (LET) [5]. Radiation energies usually range from 10^3^–10^6^ eV (KeV–MeV) [1,5]. Higher-energy radiation can transfer greater amounts of energy to matter, but the interaction probabilities are described by a complex function that depends on energy and LET [1].

As stated above, LET is an inherent characteristic of each radiation type. LET is the amount of energy transferred per unit length traveled through matter (e.g., KeV/µm) [1]. The quality factor (QF) of is defined as the ratio of the damage (ion pairs) produced by the absorption of 1 Gy of that specific form of radiation compared to the amount of damage caused by absorption of 1 Gy X-ray irradiation [13]. Quality factors range from approximately 1 (the same as 1 Gy X-ray damage) to >20 [13,14]. Radiation with QF = 1 is associated with the formation of 6–8 ion pairs when passing through 1 µm of biological tissue. Radiation with QF = 1–2 is considered to be low LET. In contrast, radiation with a high LET has a higher QF, with the induction of higher biological damage. For instance, a QF of 20 causes heavy biological damage, producing >4000–9000 ion pairs per µm [13].

X-rays and gamma rays (both types of high-energy electromagnetic waves, or photons), and beta particles (produced during radioactive decay or in linear accelerators) are characterized by variable energies, but with low LET. Artificially produced X-rays typically have energies measured in the KeV–MeV range, and gamma rays typically have energies in the MeV range [1]. X-rays and gamma rays have similar interactions with matter and similar *patterns* of energy deposition, with QF = 1 regardless of the energy [1,13]. However, the depth of penetration of photons is related to their energy levels. Lower-energy X-rays display reduced tissue penetration due to the deposition of their energy (loss of energy) along their pathway through matter. When their energy is depleted, their transit is complete [1]. In contrast, higher-energy gamma rays achieve deeper tissue penetration before their energy is depleted. Beta particles typically have energies of 0.5–5 MeV, and can travel several millimeters through tissues [13]. The relatively high energy of beta emissions allow them to have a deeper penetration in water and tissues, but their QF is 1 due to the pattern of energy deposition and distribution of ion pairs in matter [5].

Alpha particles (^4^He nuclei), high-energy protons (present in cosmic rays and proton therapy), and neutrons (usually only produced by nuclear fission reactions) are high-energy radiation species with high LET [5,15]. Alpha particles originating from radioactive decay generally have energies of 4–8 MeV, QF = 20, and can travel ~40 μm through tissues. Despite their relatively high energy, the size and charge of alpha particles limits the distance they can travel through air and tissue, and they rapidly lose energy when passing through matter [5]. High-energy protons can have a range of energies ~10–250 KeV, QF = 10 [16,17]. Slow neutrons may have energies of <10 KeV, QF~3, but fast neutrons are often >10 KeV with a quality factors of up to 10 [13]. The depth of tissue penetration by protons and neutrons varies, with lower energy particles in general having lower tissue penetration depths.

Relative biological effectiveness (RBE) is a measure of a radiation’s intrinsic biological effects (usually to compare the induction of cancer cell death or loss of clonogenicity). An additional measure, Gray Equivalents (Gy-Eq), was established in the early 21st century to provide a means to compare non-cancer, “deterministic effects” of different types of radiation, including tissue reactions such as skin burns [18]. RBE is measured on a scale with the biological effects of 250 keV photons or ^60^Co gamma radiation set equal to 1 [19,20,21]. The specific RBE of a radiation emission is determined by a variety of characteristics of the radiation, including the LET, the dose and dose rate, and the energy of the radiation, as well as characteristics of the target, including the radiation sensitivity or resistance of the tissue or cell type [20,22,23]. High-LET radiation, with more densely ionizing capacity, has higher RBE compared to low-LET radiation [24].

The improved understanding of the aspects of radiation that can be controlled (total dose, dose rate, and area of exposure) together with understanding of the innate qualities of radiation (energy, LET, RBE, and depth of tissue penetration) have allowed the development of radiation for medical applications.

## 2. Medical Applications for High- and Low-LET Radiation

**Figure 1:**
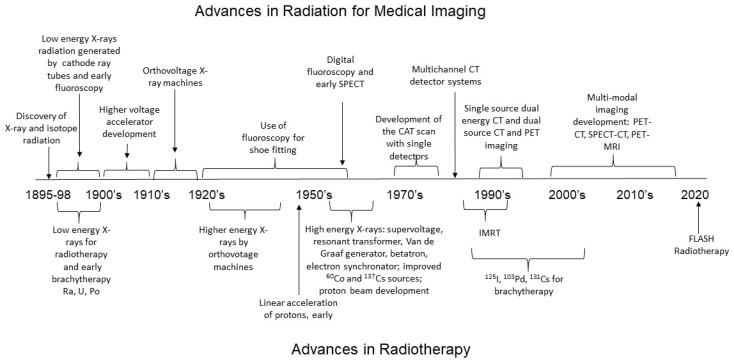
Timeline of advances in radiation for medical imaging and radiotherapy. The discovery of radiation led rapidly to its use for imaging in medicine and its use in cancer therapy. CT—computerized tomography; MRI—magnetic resonance imaging; PET-photon emission tomography; SPECT—single-photon emission computerized tomography.

On 28 December 1895, Wilhelm von Rȍntgen submitted the first radiographic image of Rȍntgen’s wife’s hand to the Proceedings of the Würzburg Physical-Medical Society [2]. This image documented the characteristic of X-rays that could pass through soft tissues but were attenuated by dense tissues such as bone and metal [1,2,4]. Within the first year of this revelation there were more than 1000 published scientific articles and 49 books on the topic of X-rays [2]. However, early radiation therapy consisted of low-energy X-ray irradiation, with very limited concepts of radiation dose regulation, no equipment for directing radiation beams, and no concept of treatment planning [25]. The development of these concepts to optimize radiation for medical imaging and cancer eradication has continued uninterrupted for the past 120 years to improve targeted delivery, reduce adverse effects, and improve patient outcomes (Figure 1).

### 2.1. Medical Imaging and the Development of Low-LET Radiation Technology

Materials exhibit different energy absorption or attenuation depending upon their electron density and effective atomic number, resulting in the absorption of photons of different energies. Materials with low atomic numbers demonstrate small differences in attenuation between high- and low-energy photons [26]. However, materials with high atomic numbers such as iodine, show large differences in absorbance [26,27]. Soft tissues of the body contain large amounts of water, resulting in very few differences in X-ray attenuation from one type of soft tissue to another. Early medical images used X-rays with mixed low energies and low tissue penetration that could only detect large differences in X-ray attenuation, for instance by bone or metals in surrounding soft tissue [2]. Later developments in improved X-ray beam generation, reduction of background and scatter, and improved detection techniques allowed the acquisition of images showing differences within soft tissues.

#### 2.1.1. Relationship between Radiation Energy and Medical Images

From the 1890s to the early 1900s, X-rays were generated using simple cathode-ray tubes, essentially positive and negative electrodes within a glass vacuum tube through which a high voltage was applied [28]. The X-rays produced by this early technology (accelerator voltage ~1000 volts) likely had mixed energies of <3 KeV, with LET of <1 [25]. Initial clinical uses of X-ray images included the diagnosis of bone fractures and the detection of foreign bodies (e.g., bullets in wounded soldiers) [2,28]. Images were often blurred due to patient movements over the time required to obtain the image and due to the scatter of the X-rays with mixed energies [29]. During this time, measurements of radiation, termed Rȍntgen dosimetry, were performed by Guido Holzknecht, utilizing changes in color of salt mixtures [25]. The semi-quantitative Holznecht unit was determined biologically as 1/5 dose of radiation that would cause erythema (reddening of the skin), an early indication of tissue injury. Later in the 1900s–1910s, medical imaging was improved by the development of higher voltage accelerators generating higher-energy X-ray photons that could penetrate thicker tissues, with greater resolution, for instance for the diagnosis of pulmonary tuberculosis [1,28].

In the 1920s, two important advances were made for medical imaging. First ionization chambers were developed, allowing more accurate measurement of radiation energy, so that the total doses of radiation delivered could be determined [25]. Second, “orthovoltage” machines were developed, operating at 200–500 KV to deliver X-rays in the range of 100–500 KeV [30]. These X-rays were referred to as “deep” X-rays due to their ability to penetrate soft tissue more efficiently [31]. This energy range is currently considered the upper limit for diagnostic radiography [31]. However, the LET of higher-energy photons remained ~1, as they maintain the same physics of energy deposition.

The development of specialized X-ray imaging techniques began in the 1950s to enhance imaging from the very low attenuation contrast between healthy and diseased tissues [32]. In some cases, *reduced* X-ray energy with increased radiation absorbance can take advantage of small differences in energy attenuation by different tissues [32,33]. For instance, in mammography, small differences in linear attenuation by adipose and glandular tissues are optimized by using relatively low-energy X-rays, mostly below 20 KeV [33]. These advances require reduced scatter, using anti-scatter grids between the tissue and the detector, and increased signal-to-noise ratio [32]. The use of phase contrast takes advantage of variations in X-ray refraction indices with tissues to help increase signal to noise [32,33]. Phase-sensitive images obtained at results showing high energies (60–500 KeV) in total dose reduction, improved image quality, and imaging in thicker tissues [32]. Finally, the use of contrast agents with different energy attenuation properties compared to biological tissues, such as iodine or barium, can be used to image specific biological structures [34].

Computed axial tomography (CAT), or computed tomography (CT), is currently used to produce over 80 million medical images in the US each year [27,35]. CAT scans, developed in the early 1970s [36], required monochromatic beams of X-rays, usually 70–140 keV [27,37]. In the 1990s technical advancements further improved X-ray generation, detectors, data acquisition, image processing, image correction, and system controls [37,38]. CT was viewed as a major advance because of the ability to detect small attenuation differences in tissue [36,39]. Low-energy photons in CT produce images where disease lesions and adjacent healthy tissues naturally have a higher attenuation difference (e.g., CT angiography, 45–55 KeV) [27]. Intermediate energies (60–75 KeV) provide contrast in soft tissues and reduce noise [27]. In advanced CT, two radiation energies (often 80–140 keV) produce additional contrast in soft tissues [27,40] [26]. Importantly, the improved contrast and detection systems do not increase radiation exposure to the patient. For instance, a dual X-ray CT results in ~2.61–2.70 mGy exposure for a chest scan, but this is not substantially different from a single-energy CT [41].

#### 2.1.2. Radiation Requirements for Real-Time Imaging of Organ Function with Fluoroscopy and Positron Emission Tomography

Fluoroscopy using continuous X-ray images combined with contrasting dyes (barium, iodine, or gadolinium) allows real-time imaging of internal organs [42,43,44]. Contrast agents administered orally or injected intravenously, intra-articularly, intrathecally, or into the uterus allow imaging of the gastrointestinal system, heart, brain, blood vessels, nervous system, or uterus and fallopian tubes [44,45]. A comparison of the radiation exposure for fluoroscopy with CT scans shows that in some cases, fluoroscopy results in higher radiation exposure. For instance, the continuous radiation exposure required for barium lower gastrointestinal imaging results in absorbed energy ~20–50 mGy [42,46], which can be higher than the total radiation required for advanced CT with contrast dye of the same area (~15 mGy) [35]. In contrast, a fluoroscopic myelogram (spinal imaging) delivers ~13 mGy, an upper GI study with barium delivers ~6 mGy, and a hysterosalpingogram results in ~1.2 mGy, compared with a spinal CT ~20 mGy, an abdominal CT image ~10–20 mGy, head or neck CT ~5 mGy, and pelvic CT up to ~10 mGy [44,47,48,49].

Positron emission tomography (PET) utilizes a radiolabeled compound taken internally from the patient and detected in tissues where it preferentially accumulates [1,50]. PET commonly uses 2-deoxy-2-[^18^F]fluoro-D-glucose (FDG), a glucose analog that is taken up and trapped inside cells. Differential uptake is based on different cellular metabolism, such as the highly metabolically active cells in tumors, or metabolic alterations in epilepsy, Alzheimer’s disease, infections, and heart disease tissues [50]. ^18^F releases positrons that upon collision with an electron release of two gamma photons of 511 KeV, with low LET [50]. The emitted gamma photons are then detected. Although the ingestion of radioactive isotopes can be a concern for patients, a comparison of ^18^F-induced DNA damage with an equivalent dose of gamma rays with matched energy (662 KeV) and total dose showed that gamma irradiation led to more DNA damage [51,52]. PET/CT was developed to further improve differentiation between normal and malignant tissues. The total radiation exposures from this combined technique are ~15–25 mGy for imaging organs such as the brain, liver, or lungs, and higher doses for the heart and bladder (~36–85 mGy) [52].

#### 2.1.3. Future Imaging Technique Development

It was at first thought that X-ray exposure to patients (and physicians) carried a low risk of injury, but it was soon found that there was a significant risk for radiation-induced tissue injury, including skin burns, tissue dysfunction, and the induction of cancer at high doses [2,4]. Concerns regarding the induction of cancer mutations by radiation exposure during medical imaging have led to the development of techniques with reduced radiation as well as the combination of radiation imaging with non-radiation techniques such as magnetic resonance imaging [1]. The development of nanoparticle contrast agents also provides another mechanism for the improvement of medical images, with the possibility of the reduction of radiation exposure due to improved signal-to-noise ratio with lower exposure times [34]. Additionally, the development of advanced detection systems, such as multi-detector CT scanning, combined with software/computer driven advances, such as machine learning (e.g., deep learning), has improved three-dimensional image reconstruction and image interpretation while lowering radiation exposures [53,54,55].

### 2.2. Radiotherapy for Cancer Treatment

In the late 1890s, Émil Grubbé, a student at Hahnemann Medical College in Chicago with an interest in electronics, had been experimenting with Rȍntgen’s X-ray cathode tube, and found that X-rays could cause significant tissue damage (in this case, to his own hand) [56]. Grubbé, in consultation with his medical professors, realized that if X-rays could cause significant damage to normal tissues, they might also be used to eradicate cancer cells [56]. This realization led to use of X-rays in January 1896 for the treatment of a patient with metastatic breast cancer [56]. By 1896, radiation for cancer eradication was described [25,57,58,59]. Currently, up to 50–60% of cancer treatments include some form of radiotherapy, contributing significantly to the improved cure rates of many cancers through the induction of lethal DNA damage [60,61]. However, because of the adverse effects of high-dose radiation to surrounding normal tissues, techniques were developed to reduce normal tissue damage, including the establishment of dosing schedules, shielding strategies, and beam targeting [57]. Unlike medical imaging, for which the goal is to minimize radiation absorption, the goal of radiotherapy for cancer treatment is the maximization of radiation exposure to cancer cells, while at the same time minimizing radiation injury to overlying and adjacent normal tissues [15,62].

#### Development of Low-LET Photon Radiation for Cancer Treatment

In the late 1800’s and early 1900s, low-energy X-rays were first used for the treatment of leukemia and breast, skin, and stomach cancers [25,56]. Treatments varied in time from 15 min to an hour, with variable differences in cancer eradication and normal tissue damage [25,56]. The X-ray generation technology (using voltages of 10–150 KV) produced low-energy photons (3–50 KeV), and although these X-rays could produce some cancer cell killing, adverse effects included severe injury to the skin due to the deposition of energy primarily in the surface tissues [1]. As a result of the low penetration of the low-energy X-rays, effective cancer eradication could only be achieved in surface tissues or when the cancerous tissue was exposed surgically [63,64,65].

From the 1920s–1950s, higher-energy X-rays became available. Orthovoltage X-rays (100–500 KeV) allowed the treatment of tumors only to a depth of 4–6 cm [30,31]. Supervoltage X-ray tubes allowed the generation 100–500 KeV X-rays [63,66], but these energies were still not optimal for deep tissue cancers [63,66]. By the late 1950′s, equipment for the generation of high-energy X-rays included the resonant transformer (up to 300 KeV electrons), the Van de Graaf generator (2–10 MeV electrons), the betatron (110 KeV electrons), and the electron synchrotron (>50–70 keV electrons) [30,66,67,68,69]. In the 1970s, linear accelerators (linacs) were introduced, capable of producing 4–30 MeV electron beams. In parallel searches for high-energy sources, ^60^Co (photon enTergy 1.17 and 1.33 MeV) and ^137^Cs (photon energy 0.662 MeV) were used to produce high-energy gamma rays. The higher-energy X-rays and photons achieved greater tissue penetration for the treatment of deep tissue cancers [63,70,71]. A direct comparison between orthovoltage and supervoltage therapies for the treatment of cancers in the tonsillar bed, pharyngeal walls, cervix, uteri and breast showed that supervoltage improved the percentage of local cancer cure [72,73] Table 1.

However, higher-energy X-ray and photon beams typically exhibited larger penumbras and large exit doses, resulting in higher exposures to normal tissues [70]. Because of this, arguments were made that lower doses of photon irradiation (~1.2–6 MeV) were preferable for the treatment of some cancers (cranial, thoracic, or lower abdominal) for the protection of normal tissues [70,81,82,83]. Additionally, the increased energy of low-LET radiation did not significantly increase the RBE. Using V79 cells in culture, it was found that compared to 4 MeV X-rays (RBE = 1), 50 MeV X-rays had an RBE = 1.1 (10% increase in RBE) [21]. Therefore, although the high-energy X-rays and gamma rays could penetrate more deeply into tissues, the cellular effects (important for cancer killing) were not increased.

### 2.3. High-LET Radiation for Cancer Treatment

As stated above, the development of X-rays and gamma radiation for cancer treatment required technological advancements to obtain higher energy, although these radiation sources are all low-LET. In contrast, the use of radioactive isotopes (with high-LET) for effective cancer eradication required increased understanding of radiation isotope half-lives, and the tissue penetration and patterns of energy deposition by different radiation types (alpha, beta or gamma) [19]. Low- and high-energy X-rays (all low LET) have slight variations in RBE, but the RBE of high-LET radiation are more variable (Table 1). Radioactive isotopes emit a specific spectrum of radiation with different energy and different half-lives, and with diverse adverse effects [19,20,75]. The increased RBE of neutrons, alpha particles, and protons over X-rays and gamma rays suggest that these radiation species would have increased efficacy in tumor cell killing [19]. Because the range of beta emissions extends for several millimeters through tissues, therapy with beta emitters can produce damage to normal tissue surrounding a targeted tumor [19]. In contrast, it has been postulated that alpha particles, with short range penetration through tissues and high LET, may offer more specific killing of tumor tissues [19]. The half-life of the radioactive isotope is also taken into consideration. Some isotopes have a half-life of hours to days which have sufficient duration, but others have half-lives of minutes, too short to produce effective treatment [19].

#### 2.3.1. Development of Brachytherapy Using Radioactive Isotopes

In 1895–1898 uranium, polonium, and radium were discovered by Becquerel and the Curies, and it was suggested that these radioactive particles could be used for cancer treatment, by direct exposure of the diseased tissues next to the radioactive particles [84]. In 1901, Henri-Alexander Danlos and physicist Eugène Bloch at St. Louis Hospital in Paris used a small tube of radium sulfate to treat tumors [84]. In 1903 brachytherapy was used for the irradiation of cancers in the skin, cervix/uterus, and prostate [29,73,85,86,87]. Brachytherapy remains a common cancer treatment for prostate, head and neck, bronchus, esophageal, breast, gynecological, rectum, anus, eye, and skin [88,89].

A number of isotopes have been investigated for cancer treatment [86,87,90]. Currently, ^103^Pd, ^125^I, and ^131^Cs are considered the most suitable isotopes for brachytherapy, due to their emission of low-energy photons, resulting in low tissue penetration to protect adjacent structures [86,91] (Table 2). Three levels of brachytherapy have been developed: high-dose rate (HDR, >12 Gy/h) [89], medium-dose rate (MDR, 2–12 Gy/h), and low-dose rate (LDR, <2 Gy/h) [88]. These treatments use the same isotopes (e.g., ^131^Cs and ^192^Ir), but utilize different amounts of isotopes. HDR and LDR treatments may be coupled with external beam radiation (EBRT) to increase cancer killing [89]. Advancements for the targeting of radioactive elements are being developed, using unique features of cancer cells (e.g., the radiolabeling of antibodies—radioimmunoconjugates) to deliver radiation to cancer cells expressing high levels of a target protein [19].

#### 2.3.2. Hadron Therapy: High-LET Radiation Beams for Cancer Treatment

It was initially thought that alpha and beta particles would be useful only for the treatment of surface or shallow cancers, due to low tissue penetration, although particle-emitting elements were first used for brachytherapy when they could be physically placed close to tumors [92]. The low-energy alpha and beta particles rapidly lost energy as they entered matter and lost momentum. However, in the mid-1940s, with greater understanding of the characteristics of radiation energy, physicists such as Robert Wilson hypothesized that accessing deeper tissue tumors could be achieved by the development of machines capable of generating high-energy particles that would not rapidly exhaust their energy during passage through tissues [92,93]. In the 1950s linear acceleration of protons, such as by the synchrocyclotron, allowed the production of proton beams of 10′s–100′s of MeV for cancer treatment [66,94].

**Figure 2:**
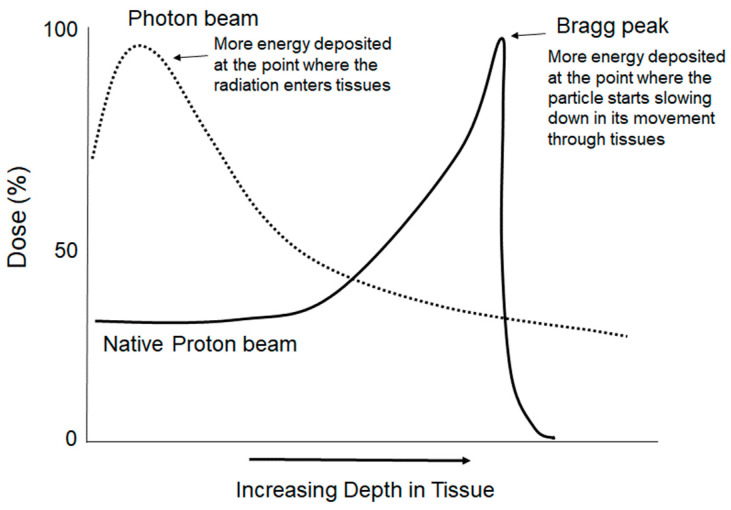
Schematic of the Bragg peak. As radiation energy traverses tissue, it distributes energy. A low-energy photon beam deposits a greater amount of energy at shallow tissue depths (dotted line). In contrast, a proton beam will deposit less energy at shallow depths. As the rate of speed of the radiation beam declines, more energy is deposited, reaching a peak release of energy at a specific depth of tissue, followed by a steep fall-off in energy deposition (solid line).

The high-energy/high-LET protons penetrate more deeply into tissues, leading to the development of intensity-modulated proton radiotherapy (IMRT) [93,95,96,97]. As charged particles traverse matter, their rate of speed declines as they lose energy. They deposit most of their energy at a specific depth within the tissue, with a peak release of energy (the Bragg peak) prior to a sharp dose fall off as the energy of the particle is depleted (Figure 2) [96,97]. Although the depth of the Bragg peak is determined by the initial energy of the proton beam, it can be spread out by a range of shifters—slabs of uniform material, usually made of plastic—to attenuate the beam to produce a series of Bragg peaks at different depths correlating to the depth of the tumor [97,98,99]. From a proton beam of 117 to 200 MeV, the range of modulation of Bragg peaks has been shown to produce up to an 8.5 cm spread [95]. A disadvantage to the use of IMRT to modulate the Bragg peaks is an increase in the scatter, with potential for increased damage to surrounding normal tissues [95].

Proton beam therapy (PBT) was initially used for the treatment of tumors where the cancers were unresectable, and when they did not respond to conventional photon radiotherapy [93,96]. PBT remains a preferred therapy for primary treatment of a number of ocular tumors, tumors localized to the central nervous system and upper digestive tract, and lung tumors [93]. This is especially due to the accuracy of radiation beam delivery at a specific depth of tissue with less scattering to surrounding normal tissue and little or no exit dose [93]. PBT is also the preferred method for re-irradiation of cancers following local or regional recurrence and for normal tissue sparing [96].

### 2.4. Techniques for Sparing Normal Tissues during X-ray Cancer Radiotherapy

With increased use of radiation for the eradication of cancer cells, it was soon determined that effective cancer treatment had to be coupled with protection of normal tissues from the collateral damage of radiotherapy [4,100]. A variety of techniques have since been developed for protecting normal tissues, including fractionated dosing, shielding, collimation, and stereotactic delivery of the radiation.

Fractionated-dose treatment, instead of single high-dose radiation treatments, was the earliest methodology developed to reduce normal tissue damage while achieving the high total dose of radiation exposure needed for cancer cell killing [25,56,101]. In the 1920s, the introduction of lower doses with rest periods in between (often 24 h) was recognized to allow time for cancer cells to be re-established into a radiation-sensitive state (See the 4 R’s, below), and also to allow recovery of normal tissues [15,60,63,102]. Current conventional fractionated radiation for the treatment of many cancers utilizes once-daily 1.8–2 Gy fractions (hyperfractionation), with one day rest period, often to achieve total doses of >60 Gy [102,103,104,105]. Other dosing regimens with varying fractionation and rest periods have been compared with the goals of optimizing effects on tumors and patient survival, and reducing adverse effects (mostly damage to adjacent normal tissues) [104,105,106].

Most recently, irradiation at ultra-high-dose rates (UHDR, ≥40 Gy/sec) in a new method termed “FLASH-radiotherapy” (FLASH-RT) have been investigated in preclinical studies and in an early clinical trial [107]. The advantage of UHDRs is an increase in the radiation fluence, defined as the number of total particles crossing a specific area. The ultra-high fluence results in a drastically increased local energy deposition [108]. Whereas high-LET causes increased energy deposition along a single track, UHDR increases energy deposition by increasing the density of tracks per volume of tissue [108]. Preclinical studies suggest that the ultra-high dose rates may maintain cancer eradication while reducing normal tissue damage using a shorter therapy time [107]. The treatment of the first patient with FLASH-RT, delivering 15 Gy in 90 ms, resulted in the eradication of a lymphoma tumor with only minimal effects on the surrounding normal tissue [109].

## 3. Cellular Effects of High- and Low-LET Radiation

Immediately upon entering tissues, radiation energy damages biological macromolecules through the rupture of chemical bonds, S-H, C-H, O-H, and N-H [110]. Radiation penetration of the nucleus causes potentially unrepairable damage to the DNA, resulting in cell death [19,111]. In contrast, irradiation of the cytoplasm is not sufficient to induce cell death [19,111]. Cell death and the loss of proliferative capacity (loss of clonogenicity) are primarily correlated with DNA damage, by the induction of double-stranded DNA breaks (DSB), single-stranded DNA breaks (SSB), apyrimidinic and apurinic sites, base modifications, and DNA–DNA and DNA–protein crosslinks [19,111,112]. Cumulative DNA damage results in unrepairable DNA fragments, chromosome destabilization, the formation of micronuclei, and the induction of toxic mutations [19,111,113,114].

A secondary effect of radiation is the generation of reactive oxygen and nitrogen species (ROS and RNS) that can also react with biological macromolecules [114]. Because water makes up a large percentage of total molecules within cells, the primary ion species produced by radiation energy are ROS, including oxygen and hydroxide free radicals [114]. Nitrogen free radicals can be generated directly by radiation, but can also be generated through the upregulation of inducible nitric oxide synthase (iNOS) activity, generating nitric oxide that can interact with superoxide to produce peroxynitrite [113,114]. The ROS and RNS cause a cascade of oxidative/nitration damage to mitochondrial and nuclear DNA, protein, and lipids [113,114].

In 1956 the first radiation–survival curve was performed for mammalian cells, examining cultured HeLa cancer cell survival as a function of X-ray dosage [115]. This was significant for providing the first demonstration of the relationship between loss of clonogenicity and increased dose of radiation [115,116]. Studies have shown that macromolecular damage is relative to the amount of energy deposition, and is thus affected by the total dose (Gy) and the LET of radiation [116]. High-LET radiation induces greater DNA damage with more complexity compared to low-LET radiation [114]. Additionally, measurements of ROS generation showed that low-LET gamma rays emitted from ^137^Cs can induce ~60 ROS per ng of tissue within 1 microsecond [114]. In contrast, ~2000 ROS per ng tissue are generated from a 3.2 MeV high-LET alpha particle, corresponding to ~19 nM ROS [114]. Thus, the potential for initial lethal DNA damage is increased by high-LET as well as the increased production of ROS and RNS with secondary toxicity [116].

### 3.1. Cancer Cell Responses to High- and Low-LET Radiation

The therapeutic goal of radiotherapy is to produce “irreparable damage in tumor cells while minimizing harm to adjacent normal tissue” [57]. Radiation oncology has been guided by four primary principles for cancer cell eradication, the “4 R’s”, first described by H.R. Withers in the 1970’s (Table 3) [15,102,117,118,119]. Sublethal DNA damage can be repaired by tumor cells at different rates, depending upon the mutations present and the repair enzymes available. Following a period of DNA repair, tumor cells again begin to proliferate and redistribute into the different phases of the cell cycle that are differentially radiation sensitive [102]. Radiation resistance is observed in late S phase and in G_0_ phase, while the greatest sensitivity is typically observed at G_2_/M phase. The resistance and sensitivity are believed to be related to the cell cycle-dependent expression of some DNA repair enzymes [118]. Cell division in a tumor can be symmetrical or asymmetrical which can result in the production of more differentiated cells, potentially with increased radiation sensitivity. The radiation resistance of tumor cells is partly attributed to the hypoxic microenvironment of the tumor (especially areas inside a bulky tumor with poor vascularization) [102,120,121]. Following radiation therapy, the reduction of tumor size (“debulking”) often occurs with normalization of the vasculature. Additionally, reduced intratumoral pressure following irradiation can result in higher oxygen levels for cells within the tumor, resulting in increased radiation sensitivity [102].

A fifth R for radiation oncology was later added when the understanding of DNA damage and capacity for DNA repair by specific types of cancer cells could be quantified: radiosensitivity [60,122]. The general tenet of radiotherapy is that cancer cells often contain mutations in enzymes that function in DNA damage recognition pathways and DNA repair pathways, rendering them more susceptible to radiation damage than the surrounding normal tissues [57]. However, clinical, preclinical, and in vitro studies showed that while some cancer cells are readily destroyed by radiotherapy, others—such as prostate and colorectal carcinomas and soft tissue sarcomas—are significantly resistant to them [15,115].

A variety of mechanisms have been identified for cancer cell evasion of radiation-induced death. A comparison of the relative sensitivity of 13 cancer cell lines to gamma irradiation showed that, generally, radiation sensitivity was proportional to chromosomal damage (correlation 0.9) [123]. However, one cancer cell line (T47D) exhibited high levels of chromosomal damage without losing clonogenicity [123]. This was attributed to the high number of chromosomes in these cells (111; DNA index > tetraploid). The genetic redundancy was hypothesized to allow tolerance to chromosome loss [123]. In other cancer cells, radiation resistance has been shown to be related to a variety of pathways and cellular characteristics, and is not always predicted by DNA repair processes [15,123].

Approaches for treating radiation-resistant cancers have included increasing the total dose of radiation exposure, increasing the radiation dose rate, and/or the use of higher LET [61]. In some cases, tumor cells resistant to low-LET radiation exhibit increased sensitivity to high-LET radiation [124,125]. High-LET radiation produces high localized energy deposition within the particle tracks during transit through tissues, with increased particle track core diameter [126]. High- and low-LET radiation induce initial DSBs with similar efficacy, but these DSBs have different qualities [127,128,129]. Single DSBs, as produced by low-LET, are usually repairable by cells [112]. However, clustered DSB (two or more lesions within one or two helical turns), produced by high-LET, are often unrepairable [112]. Electron microscopy and immunofluorescence showed that high-LET radiation induces more complex and closely clustered DNA lesions (as many as 500 DSB per µm^3^) [125,126,130]. In comparison, low-LET radiation induces a low rate of clustered DNA lesions [112,131]. Additionally, high-LET lesions require more time for repair with lower fidelity repair, resulting in more mutations, chromosomal aberrations, and chromosomal instability [125,126,127]. The relative sensitivity of cells to high and low LET has been hypothesized to be a function of potentially lethal and unrepairable breaks in the DNA [119,124].

### 3.2. Normal, Non-Cancer Cell Responses to High- and Low-LET Radiation

In contrast with cancer cells, the sensitivity of normal (non-immortalized, non-transformed) cells to radiation is related to the ability of the cell to repair DNA breaks [62]. Normal cells are generally sensitive even to low-dose radiation, and the dose–response curves for radiation-induced cell death are steep, with small increases in radiation dose having large effects on cell survival [62,124]. As evidence of the relation of DNA damage to loss of clonogenicity, cells from patients with ataxia telangiectasia, which contain mutations in DNA repair enzymes, are extremely radiation-sensitive [124]. Normal cells are also more sensitive to high-LET radiation [132]. Exposure of contact-inhibited (G_0_) diploid human fibroblasts to either 5 Gy gamma irradiation or 1.25 Gy (low LET) of 1 GeV/nucleon ^56^Fe particles (high LET) resulted in roughly equivalent RBEs (10% survival) [132]. The three irradiations also resulted in similar numbers of chromosomal aberrations, but with increased DNA damage complexity with high LET [119,124,132]. Further studies indicated that increased sensitivity to radiation in normal cells is affected by defects in DNA repair pathways and cell cycle checkpoints, genes that regulate apoptosis, and genes for the reduction of oxidative stress, inflammation, and fibrosis [62,133].

As stated above, the “4 R’s” apply to the use of radiation for cancer cell killing, but they can also be applied for sparing normal tissues. For instance, with regard to fractionated dosing, it is desirable to allow time for sublethal damage to be repaired between exposures to reduce the adverse effects of radiotherapy [134]. Repair in normal tissue has to be countered by not allowing too much time for tumor cell proliferation [134]. Thus, repair and repopulation are most important for sparing normal tissue, but redistribution/reassortment and reoxygenation are most important for tumor cell killing [134].

## 4. Signal Transduction by High and Low LET

DNA damage (repairable or unrepairable) leads to the rapid regulation of potent signaling pathways, with biological outcomes dependent upon a variety of cellular characteristics. In cancer cells, possible outcomes include repair and re-entry into the cell cycle, apoptosis, necrosis/necroptosis, autophagy, and accelerated senescence [112,114,123,133,135,136,137,138]. Many primary cells have been shown to primarily undergo accelerated senescence from low-LET radiation, but apoptosis can be observed at higher total doses of low-LET radiation (>50 Gy) [127,136]. Studies of cancer and normal cells have provided evidence that the biological “decisions” for non-survival versus survival are guided by signaling pathways, activated downstream of the initial DNA damage response, that are influenced by numerous characteristics specific to each cell type and its environment [114,123,133,135,136,138,139,140,141].

### 4.1. Pathways for DNA Repair

DNA damage, especially DSB, rapidly induces the DNA damage response (DDR) pathway [133,142]. The primary pathways of DBS DNA repair are homologous recombination repair (HRR), non-homologous end-joining (NHEJ), and alternative end-joining (alt-EJ) [133,142,143,144]. These three pathways involve different initiating proteins that recognize DSB and different downstream enzymes (Table 4). Of the three repair pathways, NHEJ is the most rapid and efficient pathway but is also the most error-prone [143]. NHEJ is fairly independent of the phases of the cell cycle for its function. In contrast, the HRR pathway is restricted to the S and G_2_ cell cycle phases, when the proteins and enzymes required are present. HRR uses the sister chromatid as a template and is the least error-prone pathway but it is also slower than NHEJ [143]. Alt-EJ, the least understood pathway, is similar to NHEJ, and can also result in deletions with microhomologies at the repair site, although it shares initiating proteins with HRR [143,145].

A number of studies have addressed the preferential DNA repair pathways activated by low- and high-LET radiation. A comparison of wild-type cells with cells lacking proteins for NHEJ showed that cells deficient for NHEJ contained an increased number of residual, unrepaired DSB following low-LET exposures [149,150]. A study of cells and mice deficient in the HRR pathway showed that there was greater sensitivity (loss of clonogenicity in cells and mortality in mice) in response to high-LET radiation than to low-LET radiation [144]. In contrast, NHEJ-deficient mice or cells had equally increased sensitivity to high- and low-LET radiation [144]. Later studies investigated the survival of wild-type cell lines, cell lines with deficiencies in NHEJ or HRR pathways following exposure to gamma rays, protons, and carbon ions [151,152]. The data showed that deficiency in DNA-PKcs (NHEJ deficient) caused increased sensitivity to all three types of radiation. However, increased sensitivity was observed in HRR-deficient cells only following carbon ion irradiation (high LET). These three studies suggest that the HRR pathway is more critical for DSB repair in response to high-LET than for low-LET radiation, but that NHEJ is required for DSB repair from high- and low-LET radiation [151].

DSB foci induced by high-LET radiation require a longer period of time to resolve. Following exposure to 0.5 Gy of either gamma rays, protons, carbon ions or alpha particles, it was observed that DSB-protein complexes persisted longer in the cell nucleus following higher LET irradiation [153]. In a similar study of 1 Gy X-ray, or carbon or iron ions, DSB foci in peripheral blood mononuclear cells were slower to resolve from high- LET radiations [154]. The dependence of high LET on HRR for DNA repair and the reduced rate of DNA repair in general suggested that high-LET radiation may reduce the efficiency of NHEJ [153,155]. A study comparing the lengths of DNA fragments produced by high- and low-LET radiation led to the hypothesis that the shorter DNA fragments produced by high-LET radiation (<40 base pairs) may preclude efficient Ku binding to the two ends of a fragment at the same time, thus reducing NHEJ repair capacity, which may result in the importance of HRR in high-LET radiation-induced DNA damage repair [156].

### 4.2. Regulation of the Cell Cycle: The Gateway for Cell Death or Accelerated Senescence

The DDR pathway coordinates signaling cascades to pause the cell cycle, regulate transcription and translation, alter metabolic functions, and modify chromatin structure [114,133,157,158]. The outcome of DDR, paused cell cycle and metabolic changes, can result in a return to normal cellular function, apoptosis or other mechanisms of programmed cell death, necrosis, autophagy, or accelerated senescence [136,159,160].

The regulation of the cell cycle occurs rapidly following radiation breakage of DNA, after the formation of initial complexes of proteins to stabilize DNA breaks. DSB are rapidly recognized and bound by three kinases, ataxia-telangiectasia mutated (ATM), ataxia-telangiectasia and Rad3-related (ATR), and protein kinase C-associated kinase (PKK) [133,148]. These proteins can regulate downstream phosphorylation and dephosphorylation events, as well as rapid changes in gene expression. These changes result in the inactivation of cycle-dependent kinases, the activation/upregulation cell cycle checkpoint proteins and of specific transcription factors to efficiently pause the cell cycle and prevent progression through G_1_/S and G_2_/M phases [114,148]. The coupling of DNA repair with the inhibition of the cell cycle is hypothesized to promote cell survival, as progression of the cell cycle in the presence of DNA damage could result in catastrophic chromosomal damage [148].

The anti-oncogene and transcription factor p53 is a key regulator of pathway activation downstream of the DDR pathway [161]. p53 protein is recruited to DSB lesions containing ATM, ATR, and phosphorylated histone H2AX (γH2AX) [131]. The specific rearrangements of phosphorylation on p53 at 18 amino acids are critical for the modulation of its downstream activity [148,154,161]. Following DDR activation by radiation, p53 is rapidly dephosphorylated (dephosphosphorylated-S37, -S46, and -T55—apoptosis), while other sites are phosphorylated (phospho-S15—cell cycle arrest) [148,161,162]. p53 has been shown to have at least 350 confirmed gene targets and over 3500 potential targets [148,154,161]. A lack of p53 protein activation was shown to result in the loss of cell cycle inhibition (at either G1/S or G2/M) and the loss of cell checkpoint protein regulation [163]. A critical target of p53 gene regulation is p21/waf 1, a potent inhibitor of cell cycle progression through its binding to cyclins CDK2, CDK1 and CDK4/6, pausing the cell cycle at G1 and S phases [164]. Following a pause of the cell cycle, by p53 and p21/waf1 activities, the cell can undergo apoptosis, accelerated senescence, or necrosis.

High-dose, low-LET radiation (50 Gy) induces p53- and p21/waf1-dependent apoptosis in normal primary pulmonary artery endothelial cells [160,165]. In lymphoblastoid cell lines, low-LET radiation also induces apoptosis through increased p21/waf1 and p53 levels, and increased p53 phosphorylation on S15 [166]. In contrast, high-LET radiation induced apoptosis without significant increase in p53 or p21/waf1 in these cells [167]. Mutations in p53 are associated with resistance to radiation-induced cell death and resistance to cell cycle regulation, especially mutations that affect p53 regulation of p21/waf1 [168]. Niemantsverdriet et al. compared p53 phosphorylation at S37 (apoptotic signaling) and S315 (fibrotic signaling) by high- and low-LET radiation, and downstream regulation of the pro-fibrotic gene plasminogen activator inhibitor 1 (PAI-1) in transformed lung epithelial cells (A549) and immortalized, non-transformed human embryonic kidney cells (HEK) [169]. The data showed that high-LET carbon ions and low-LET photons induced similar levels of p53 phosphorylation at S315 and similar levels of PAI-1 regulation. However, carbon ion radiation induced higher apoptosis, correlating with increased phosphorylation of p53 at S37 [169].

As stated above, apoptosis induced by high-LET radiation was found in some cancer cells to be independent of p53 signaling [170,171,172]. High-LET radiation was found to activate caspase-9 in the presence of mutated p53, through activation of the death receptor pathway and/or through the induction of mitochondrial stress [170,171]. In this case, caspase-3 activation was downstream of caspase-9 [171,172]. The bypassing of p53 signaling for the induction of apoptosis is specific to high-LET radiation, and was hypothesized to be related to increased activation of PARP1 and potentially due to increased damage to the mitochondria [171]. Additionally, cancer cells can undergo regulated cell death (usually apoptosis) in response to mitotic catastrophe [173]. Mitotic catastrophe is a mechanism of cell death in cells that are unable to complete mitosis due to excessive DNA damage, mitotic machinery defects, or failure of mitotic checkpoints [174]. Because many cancer cells are deficient in cell cycle checkpoints, they may enter mitosis in the presence of unrepaired DNA damage [173]. These mechanisms of cell death are particularly important for the destruction of cancer cells that often display mutations in p53. These added mechanisms for the induction of apoptosis in cancer cells provide an increased rationale for the use of high-LET radiation over low-LET radiation for the treatment of specific cancers with the induction of complex DNA damage that may not be repaired.

Accelerated senescence is another major outcome following radiation exposure. Replicative senescence is defined as the process by which normal cells reach the end of their proliferative capacity [175]. Normal cells are believed to undergo a limited number of cellular divisions, ~50, which is termed the Hayflick limit [175]. Replicative senescence has a number of characteristics: permanent exit from the cell cycle (often in G_1_ or G_2_ phases); sustained upregulation of cell cycle checkpoint proteins; shortening of telomeres; alterations in morphology, often broadening and flattening; alterations in cell–cell contacts; increased mitochondrial oxidative metabolism; and the secretion of an altered variety of proteins, especially pro-inflammatory cytokines (termed the senescence-associated secretory phenotype) [141,176]. DNA damage and oxidative stress can cause normal cells to enter senescence prematurely (accelerated senescence) [136,160,176]. Downstream of DDR and p53/p21/waf1-induced cell cycle arrest, the activation of AMP-dependent kinase (AMPK), mammalian target of rapamycin (mTOR), and phosphatidylinositol 3-kinase (PI-3K) can signal senescence in normal cells after radiation [136,160,165,176,177]. An additional pathway for the induction of accelerated senescence in normal endothelial cells involves oxidative damage to the mitochondria, with damage to respiratory complex II [178].

Cancer cells, through a variety of mutations and constitutive signaling pathway activations, often evade senescence processes [179,180]. However, both low- and high-LET radiation induces senescence in some types of cancer [177,179]. Senescence in human uveal melanoma 92–1 cells was more effectively induced by heavy ions than by low-LET radiation, correlating with more complex DNA damage and lack of repair [181]. Interestingly, X-rays also induced unrepairable DNA damage leading to senescence, but this DNA damage was specifically localized to the telomeres [181]. Low-dose, low-LET radiation (≤10 Gy) primarily induces senescence in normal cells in culture and in vivo [136,160,165,177,182]. Interestingly, high-LET radiation induced more pro-inflammatory cytokine secretion (a marker of senescence) than low-LET radiation in normal human bronchial epithelial cells [183]. Accelerated senescence in the bone marrow of mice was also observed to be higher in response to high-LET ^56^Fe ions than for protons (low LET) [182].

Ionizing radiation has been shown to induce necrosis in some cell types, although the induction of apoptosis and senescence is more commonly observed [136,160,184]. Electron microscopy imaging of peripheral blood leukocytes showed that at low-doses of high- or low-LET radiation, apoptosis was generally observed [185]. Necrosis was detected after high-dose, low-LET radiation (20 Gy), but not after high-dose high-LET radiation [185]. Necrosis is observed in whole tissues following radiotherapy for cancer treatment, but this may be an effect of loss of normal vascular tissue support or other changes in tissue structure, and not a direct induction of necrosis by the radiation [186,187,188,189].

### 4.3. Regulation of the Protein Degradation, Endoplasmic Reticulum Stress, and the Unfolded Protein Response Pathway

As discussed above, the higher energy release in a shorter area by high-LET radiation results in more complex DNA damage. Studies in vivo and in vitro following low- and high-LET radiation show a variety of protein modifications, including carbonylation and 4-hydroxynonenal (HNE) adducts [110,114,190,191,192,193,194]. Increased protein modification was observed with an increased total dose of radiation and with increased LET [193,195]. Protein oxidative carbonylation is irreversible, and HNE adducts are partially irreversible. The presence of these modified proteins leads to endoplasmic reticulum stress [196,197].

The removal of oxidized and modified proteins requires pathway activation leading to proteasomal or lysosomal degradation [196,197,198,199]. The proteasomal system is primarily used for the degradation of soluble proteins, and involves specific ubiquitination of the target protein followed by recognition and proteolysis by the 26S proteasome [200]. Deubiquitylating enzymes (DUBs) can remove ubiquitin from a protein, resulting a protein’s stabilization prior to delivery to the proteasome, making this pathway partially reversible. Unfolded proteins in the endoplasmic reticulum (ER) can induce ER stress responses. These proteins can be removed via the ER-associated protein degradation pathway (ERAD), involving the export of proteins from the ER, assisted by their ubiquitination and, their delivery to the proteasome [201]. ER stress can also induce the unfolded protein response (UPR) pathway, which is considered to be the pathway required for the removal of protein aggregates or proteins are not efficiently processed via the ubiquitin pathway [201]. The UPR pathway is initiated by the detection of unfolded proteins in the ER through three unfolded protein sensors: double-stranded RNA-activated protein kinase (PRK)-like ER kinase (PERK), activating transcription factor 6 (ATF6), and inositol-requiring enzyme 1 (IRE1) [201]. These sensors activate different downstream signaling pathways to regulate chaperonins, redox homeostasis proteins, protein secretion, lipid biosynthesis, and cell death programs [201]. Activation of the UPR increases the delivery of proteins to the lysosome for degradation [201]. The UPR system has cross-talk with autophagy, which involves the encapsulation of large portions of the cytoplasm in isolated membrane compartments followed by fusion with the lysosome for large-scale degradation [201]. Activation of autophagy may lead to either cell death or survival [202]. Depending upon the level of protein unfolding and the oxidation, some or all of these pathways may be activated by radiation [202,203,204].

Protein ubiquitination and proteasome activity are activated by low- and high-LET radiation [139,205,206,207]. Proteomic studies in normal skin fibroblasts showed that increasing doses of high-LET radiation (0.2–2 Gy) as well as increasing from low to high LET (12.6 keV/µm–31.5 keV/µm) resulted in *fewer* protein changes at 4 h post-irradiation [207]. Gene ontology analysis of the protein changes characteristic for all LET radiations showed the regulation of pathways for RNA metabolic processes (RNA splicing, destabilization, and deadenylation) and proteasome pathways [207]. A study of gene expression in mouse blood following 3 Gy exposure to X-rays, 0.75 Gy neutrons or to mixed field photon/neutrons (total 3 Gy) showed that genes involved in protein ubiquitination pathways were significantly upregulated by all conditions [208]. In another study, blockade of proteasomal activity using N-carbobenzyoxyl-L-leucyl-L-leucyl-L-leucinal, lactacystin, or celastrol protected peripheral blood mononuclear cells from apoptosis [209]. The increased survival correlated with higher levels of Mn-superoxide dismutase (MnSOD), catalase, heat shock protein 70 (Hsp70), and glutathione S-transferase pi (GST-pi), in part through antioxidant activity. Thus, in primary cells, blockade of the proteasomal pathway was demonstrated to improve normal cell survival, potentially through increased antioxidant activity.

Besides the process of removal of damaged proteins, the proteasome pathway is also required for maintenance of protein ratios for homeostasis [210,211,212,213,214]. The ubiquitin proteasome pathway, involving both ubiquitin ligases and DUBs, is important for the DNA repair pathways for the regulation of the correct ratios of specific cellular DNA repair proteins [210]. An siRNA DUB knockout study showed that the ubiquitin proteasome pathway was required for cancer cell survival following exposure to high-LET radiation, but was not required following low-LET radiation [215]. Ubiquitin-specific protease 6 (USP6) and ubiquitin-specific protease 9X (USP9X) were required for DNA repair and survival in HeLa and oropharyngeal squamous cell carcinoma cells following high-LET radiation [215,216]. Experiments showed that USP9X depletion using siRNA did not interfere with cell cycle progression or complex DNA damage repair, and likewise did not affect levels of apoptosis, autophagy, or senescence [216]. Instead, USP9X depletion was shown to impact centrosome stability, leading to chromosomal aberrations [216]. Therefore, in these studies, blockade of ubiquitination and the proteasomal pathway led to increased cell death in cancer cells. The effect of the ubiquitin proteasome pathway in normal cells following radiation has not yet been elucidated.

ER stress, the UPR, and autophagy are also activated by both high- and low-LET radiation [203,204,217,218]. The fate of cells following the activation of these pathways depends on the duration and the degree of the response [121,202]. Studies in osteosarcoma and lymphoma cells showed that increased LET induced higher levels of UPR and autophagy [139,218]. ER stress and autophagy were activated by high-LET (I^125^) radiation in human esophageal squamous cell carcinoma [219]. Knockdown of PERK pathways downstream of ER stress in the carcinoma cells led to decreased autophagy and decreased cell survival [219]. In another study of high-LET ^56^Fe (500 MeV/n) ion radiation, ER stress led to the activation of PERK and autophagy [217].Regulation of lysosomal activity is critical for cell survival following radiation exposure [220,221]. High-doses (50 Gy) of low-LET (X-ray) radiation in normal lung endothelial cells activate ER stress response [160]. In these cells, blockade of ER stress and autophagy with salubrinal reduced apoptosis by ~50%, with no effect on senescence [160]. Together, these data suggest that there may be differential responses of normal and cancer cells to radiation-induced protein oxidation and/or unfolding, but further research is needed to determine the effects of LET and radiation doses for these effects [202].

## 5. Protein Expression and Gene Transcription by High- and Low-LET Radiation

As described above, high- and low-LET radiation differentially affects cell signaling events and the biological outcomes in cells. Global protein analyses and global genome expression analyses have been used to identify differences in cellular responses to high- and low-LET radiation. The protein expression, transcription factor regulation, and gene regulation were obtained using a variety of technologies with normal human cells and cancer cells. Transcriptomic and microarray studies revealed that although many of the same genes were regulated in response to low- and high-LET radiation, some important differences were obtained when comparing cancer and normal cells [222,223,224].

### 5.1. Alterations in Protein Levels with Low- and HIGH-LET Radiation

Using a proteomics approach, Wang et al. investigated the effects of low- and high- LET on protein expression in mouse embryonic fibroblasts (MEFs) [207]. MEFs were exposed to carbon ion beams with LET values of 12.6 or 31.5 KeV/μm, and proteomic alterations were examined 4 h after exposure. The profiles of complex changes in protein levels showed distinct patterns in each radiation group. Surprisingly, there were *reduced* numbers of proteins changed at high-dose, high-LET radiation. Gene ontology (GO) analysis showed that the highest numbers of altered protein were involved in RNA metabolic processes and proteasome pathways. Interestingly, both high-LET exposures induced increased collagen expression (including the Col1a1) and fibronectin, suggesting that the cell modifies the extracellular matrix in response to radiation and/or redox changes [207].

### 5.2. Regulation of p53 and NF-κB Transcription Factors

Initial studies examining changes in gene expression following radiation exposure focused on transcription factor activation. Many of the response pathways for inhibition of the cell cycle, regulation of DNA repair, and regulation of apoptosis are p53-regulated. As stated above p53 is rapidly recruited by DDR pathways to activate genes in pathways involving the cell cycle, growth arrest, apoptosis, etc. Additionally, NF-κB can be activated through the classical pathway by radiation-induced ROS [225,226], and through an atypical, genotoxic stress pathway, downstream of the DDR pathway [138,226,227]. In general, NF-κB activation requires proteasomal degradation of IκB, cytoplasmic inhibitors of NF-κB. NF-κB regulates antioxidant and inflammatory gene expression and can affect cell survival from genotoxic stress [226].

A transcriptomic study of normal peripheral blood mononuclear cells showed that p53 was the primary transcription factor activated at 8 h following exposure to 1 Gy carbon or iron ion radiation or 1 Gy of X-ray irradiation, albeit with different kinetics [154]. Similar results were also found using normal fibroblasts, human mesenchymal stem cells, and human bronchial epithelial cells exposed to gamma radiation and high-LET radiation (^125^I radiation, iron, or silicon ions), where genes regulating cell cycle and DNA damage were regulated comparably, likely downstream of p53 activation [228,229,230]. These studies used similar doses of radiation, 0.5–1 Gy, although the dose rates varied.

In contrast, studies suggest that the activation of NF-κB is more dependent upon the total dose and LET of radiation exposure, as well as on the cell type examined [154,231,232]. A study of HEK cells showed that NF-κB was activated at 4 h by ~1 Gy of high-LET radiation, but required 16 Gy of X-rays for activation at this time point [226]. In another study of HEK cells, heavy ions with a LET of 100–300 keV/µm displayed a nine-fold higher potential for NF-κB activation compared to X-rays, with maximal activation ~16 h [231]. In contrast, a study of gene regulation in human and murine lymphoma cells indicated that a majority of the significant gene expression induced by either high- or low-LET radiation (5 Gy for both) was likely downstream of NF-κB activation [139]. Interestingly, NF-κB activity was found to be *reduced* by low-dose (<2 Gy), low-LET radiation in macrophages and in tumors in vivo [233,234]. This suppression was attributed to reduced inactivation of IκB by inhibition of the proteasome [154,234].

### 5.3. Comparison of Low- and High-LET Radiation Using Genomic Analysis in Cancer Cells

Microarrays and global gene expression analysis showed that in many cases high- and low-LET radiation exposures in cancer cells resulted in large overlaps in gene expression, with similarities in pathways for regulation of cell cycle, proliferation, apoptosis, and inflammation [222,226,235,236,237,238,239,240,241]. Differential gene expression in oral squamous cell carcinoma (OSCC) was determined at 4 h after exposure to X-rays (2, 4, or 6 Gy) or carbon or neon ions (1, 4, 7 Gy) using microarrays [222,223,236,242] (Table 5). All doses of carbon irradiation significantly altered 85 genes in OSCC, while X-ray irradiation significantly altered only 30 [223,242]. GO analysis indicated that high-LET radiation differentially regulated genes involved in cell death, cell cycle, motility, cancer, and tumor morphology [222]. Especially of interest was the modulation of the transforming growth factor signaling and tumor necrosis factor signaling pathways by high-LET radiation [223,235]. A comparison of gene expression changes in radiation resistant and radiation sensitive OSCC showed that radiation resistance was associated with the regulation of pathways for inflammation, proliferation, apoptosis, extracellular matrix modification, and cell cycle regulation [222,223,242].

In a separate study, Sertorio et al. exposed human (BL41) and murine (J3D) lymphoma cell lines to 5 Gy of proton radiation or X-ray radiation, and used RNAseq technology to determine differential gene expression at 24 h (GSE143550) [139]. Principal component analysis of total RNA from both species showed that the proton and X-ray exposures resulted in divergent patterns of RNA expression that differed from each other and from control. A common theme for *both* high- and low-LET radiation was the modulation of pathways for energy metabolism, including fatty acid beta-oxidation using acyl-CoA oxidase, nucleotide catabolic processes, ATP biosynthetic processes, and energy homeostasis. To identify the top regulated genes by X-ray versus proton radiation, we re-analyzed the data from Sertorio et al., utilizing a more stringent cutoff ≥1.5-fold change and FDR < 0.05, using the count normalization and differential expression analysis tool, edgeR [243] (Table 5). This more stringent analysis showed that X-ray exposure of the human lymphoma cell line resulted in the significant increase of six genes, all potentially regulated by NF-κB and with functions in inflammation or immune modulation and roles in cancer progression.

Regulation of cytokines, chemokines, and other inflammatory agents (with both pro- and anti-inflammatory activities) as well as antioxidant enzymes and factors have been observed in response to both high- and low-LET radiation in vivo and in vitro. As stated above, cytokine production is characteristic of the senescence-associated secretory phenotype [244], but cytokine production may also provide autocrine signaling for responses to oxidative stress, induction of apoptosis or cell survival, cell motility, and extracellular matrix modification in cancer cells that express cytokine receptors [15,227,240,245,246].

### 5.4. Comparison of Low- and High-LET Radiation Using Genomic Analysis in Normal Cells

Several microarray and genomics studies examined gene expression in normal, non-cancer cells exposed to low- and high-LET radiation (Table 6). For many normal cell types, high- and low-LET radiation resulted in distinct patterns of gene regulation, but with overlapping gene ontology for apoptotic signaling, DNA repair, and cell cycle regulation [154]. In most cases, analysis of genes to determine transcription factor activation identified p53 as the most highly activated, by more than 10–20-fold compared with other predicted factors. Inflammatory responses, often regulated through NF-κB, were more strongly activated by high-LET radiation in most, but not all, cell types [154,226,228,229,230].

Microarrays were used to examine gene expression in human fibroblasts exposed to 1 Gy gamma and ^125^I radiation at 2 h [228] This study found an overlap in two-thirds of the differentially expressed genes (2303 from high LET, and 2163 in low LET) [228]. Gene ontology analysis showed upregulation of pathways for oxidative phosphorylation, apoptosis, and cell cycle, and a downregulation in genes in translation elongation, negative regulation of cell growth, and protein targeting [228]. Another microarray study examined the effects of 1 Gy X-ray and ^56^Fe ion radiation on human mesenchymal stem cells at 24 h [229]. Similar levels of cell cycle arrest (G_1_/G_0_) were induced by both types of radiation at 0.1 and 1 Gy. There were 81 genes commonly regulated by both types of radiation; gene ontology showed that these were grouped in cell cycle arrest, the DNA damage response, and other DNA interaction pathways. ^56^Fe irradiation additionally showed regulation of pyrimidine and purine metabolism [229].

Whole-genome expression arrays showed that human bronchial epithelial cell line (HEBC3KT) responded similarly to high- and low-LET radiation, although the kinetics differed [230]. Cells were exposed to 1 and 3 Gy gamma rays or 0.5 and 1 Gy ^56^Fe, or ^26^Si particle irradiation and gene expression was examined between 1–24 h [230]. Although there were minor differences in individual gene expression from the three types of radiation, gene ontology analysis showed similarities in pathway regulation. Predominant changes were observed in pathways for p53-dependent cell cycle, DNA replication/recombination/repair, cell proliferation, and apoptosis regulation. Low-LET radiation showed additional activation of genes involved in the inhibition of angiogenesis. The high-LET radiation groups additionally displayed regulation of acute phase response signaling. Notably absent in this cell type were NF-κB-regulated inflammatory genes.

## 6. Summary and Conclusions

The utilization of radiation is expanding, in medicine for imaging and disease treatment and for a variety of industrial and energy purposes. The specific use of high- and low-LET radiation can take advantage of the energy characteristics of these types of radiation. Additionally, the safety controls must also be specific for each type of radiation. Research has shown that the biological effects, signal transduction, and gene regulation elicited by high- and low-LET radiation differ. Additionally, the response to the radiation LET is in part a function of the cell type, with differences observed between normal and cancer cells. Findings indicate that while both low- and high-LET radiation activate NHEJ DNA repair activity, efficient repair of high-LET radiation requires HRR. Both low- and high-LET radiation activate p53 transcription factor activity in most cells, but high-LET activates NF-kB transcription factor at lower radiation doses than low-LET radiation. The preferential transcription factor activation is reflected in downstream gene regulation. Future studies of both radiation-sensitive and radiation-insensitive cancer cells and of normal cells are needed for a more complete understanding of biological responses to high- and low-LET radiation. Understanding differential transcription factor activation and gene regulation is essential for the advancement of radiation-related therapies, as identification of the modulated genes and pathways responding to specific radiation types could give rise to novel countermeasures or synergistic treatment strategies.

## Figures and Tables

**Table 1:** Relative biological effectiveness of different types of radiation.

Radiation	RBE	Energy Range	References
Alpha particles	4–20	3.2–9 MeV	[19,74,75]
Beta particles	1–3.5	0.019–1.7 MeV	[20]
Slow neutrons	~2.5–20	~10–100 KeV	[18,76,77,78]
Fast neutrons	~5–20	0.1–3 MeV	[18,76,77,78]
Protons	~0.89–3.1 *	50–1000 MeV	[22,23,79,80]
Gamma rays	~1	1.2–6 MeV	[19,20]
X-rays	~1–1.1	200–50 MeV	[19,20,21]

RBE—relative biological effectiveness. * RBE is thought to be in part dependent on radiation sensitivity of the tissue examined.

**Table 2:** Radioactive isotopes for brachytherapy.

Isotope	Radiation Type	Half-Life
^103^Pd	21 KeV gamma *	17 days
^125^I	27–35 KeV gamma *	60.25 days
^131^Cs	29.5–33.5 KeV gamma *	9.7 days
^192^Ir	206–485 KeV gamma *	74.17 days
^198^Au	314 KeV beta, 412 KeV gamma *	2.7 days
^226^Ra	47–2450 KeV gamma	1600 years

* most common emissions.

**Table 3:** The 4 R’s and the 5th R.

“R”	Definition
Repair	Sublethal DNA damage repair
Redistribution/reassortment	Redistribution of tumor cells into phases of the cell cycle
Repopulation	Tumor cell proliferation, symmetrical or asymmetrical division
Reoxygenation	Normalization of the hypoxic tumor microenvironment
Radiosensitivity	Susceptibility to radiation-induced cell death due to chromosome number alterations or mutations

**Table 4:** Comparison of enzymes in different DNA repair pathways.

Repair Pathway	Initiating Proteins	DNA Repair	References
HRR	MRN/CtIP	RPA/BRCA_2_/RAD51	[133,144,146,147]
NHEJ	Ku70/80	DNA PKcs/XRCC4/Artemis/Pol µ or Pol γ	[133,144,147]
Alt-EJ	MRN/CtIP	DNA ligase III/PARP1/pol θ	[133,147,148]

Alt-EJ—alternative end joining; ATM—ataxia telangiectasia mutated; BRCA2—breast cancer 2; CtIP—CtBP-interacting protein; DNA-PKcs—DNA dependent protein kinase; HRR—homologous recombination repair; MRN—Mre11-Rad50-Nbs1; NHEJ—non-homologous end-joining; PARP1—poly (ADP-ribose) polymerase 1; pol—polymerase; RAD51—radiation 51; XRCC4—X-ray repair cross-complementing 4.

**Table 5:** Comparison of gene expression in cancer cells after low- or high-LET radiation.

Cell Type	Radiation	Time	Genes	References
Oral squamous cell carcinoma (High v Low LET) *	X-ray (2, 4, 6 Gy) LET ~ 1 KeV/µm ^12^C (290 MeV/n) (1, 4, 7 Gy) LET = 75 KeV/µm ^22^Ne (400 MeV/n) (1, 4, 7 Gy) LET = 75 KeV/µm	4 h	↑TGFBR2, ↑SMURF2, ↓BMP7, ↑CCND1, ↑F2F3, ↑SPHK1	[222,236]
Lymphoma	X-ray (6 MeV, 5 Gy) LET ~ 1 KeV/µm	24 h	↑CCL5, ↑ CCL17, ↑CCL22, ↑GNG8, ↑HMOX1, ↑IL32	[139]
Lymphoma	Proton (129.3–148.2 MeV/n (5 Gy) LET = 3.5 KeV/µm	24 h	↑CCL5, ↑CCL17, ↑CCL22, ↑GNG8, ↑HMOX1, ↑IL32, ↑LRK2, ↑TNF	[139]

LET values are as reported in the reference. * Genes are shown that were increased in high LET vs. low LET. ↑—upregulation; ↓—downregulation.

**Table 6:** Comparison of gene expression in normal cells after low- or high-LET radiation.

Cell Type	Radiation	Time	Genes	Reference
Peripheral blood mononuclear cells ^1^	Gamma (250 keV, 1 Gy) LET ~ 1 KeV/µm	8 h	↑PCNA, ↑GADD45A, ↑ASTN2, ↑FDXR, ↑RPS27L, ↑VWCE, ↑PTPN14, ↑EDA2R, ↑CDKN1A, ↑IKBIP, ↑ANKRA2	[154]
	^12^C (114.6–158.4 MeV/n, 0.25, 1 Gy) (1, 4, 7 Gy) LET = 60–80 KeV/µm ^56^Fe (1 GeV/n, 0.25, 1 Gy) LET = 155 KeV/µm	8 h	↑PCNA, ↑GADD45A, ↑ASTN2, ↑FDXR, ↑RPS27L, ↑VWCE, ↑PTPN14, ↑EDA2R, ↑CD80, ↑BCL2L1	[154]
Human bronchial epithelial cells ^1^	Gamma (662 KeV 1,3 Gy) LET = 0.2 KeV/µm	1, 4, 12, 24 h	↑CDKN1A, ↑CCNA1, ↑ BTG2, ↑TRIM22, ↑ INPP5D, ↑GLUL, ↑THBS1, ↓SH3GL3	[230]
	^56^Fe (1 GeV/n, 0.5, 1 Gy) LET = 150 KeV/µm ^28^SI (1 GeV/n, 0.5, 1 Gy) LET = 44 KeV/µm	1, 4, 12, 24 h	↑CDKN1A, ↑CCNA1, ↑ BTG2, ↑TRIM22, ↑INPP5D, ↑GLUL, ↓APH1B, ↑BLNK, ↑PLD1, ↑PLD3	[230]
HEK ^2^	X-rays (4, 8 Gy, 200 keV) LET ~ 1 KeV/µm	6 h	↑TNF, ↑CXCL1, ↑CXCL2, ↑CXCL8, ↑CXCL10, ↑CCL2, ↑CD83, ↑NFKB2, ↑VCAM1, ↑NFKBIA, ↑BIRK3, ↓MAP2K6	[226]
	^22^Ne ions (4 Gy, 80 MeV/n) LET = 92 KeV/µm	6 h	↑TNF, ↑CXCL1, ↑CXCL8, ↑CXCL10, ↑CCL2, ↑CD83, ↑NFKB2, ↑NFKBIA, ↑VCAM1	[226]

LET values are as reported in the reference. ^1^ Short list of regulated genes shown here. ^2^ Genes identified by NF-κB pathway focused PCR target gene arrays. ↑—upregulation; ↓—downregulation.

## Data Availability

Not applicable.
